# Supplementary material for: Effectiveness of dismantling strategies on moderated vs. unmoderated online social platforms
Source: Sci Rep. 2020 Sep 1;10:14392. doi: 10.1038/s41598-020-71231-3 (PMC7462854; doi:10.1038/s41598-020-71231-3)
Supplement: Supplementary file 1 — Supplementary information. [file 41598_2020_71231_MOESM1_ESM.pdf]

Supplemental Material for *Effectiveness of dismantling strategies on moderated vs. unmoderated online social platforms*

Oriol Artime[1], Valeria d'Andrea[1], Riccardo Gallotti[1], Pier Luigi Sacco[2,3,4], Manlio De Domenico[1]

[1]CoMuNe Lab, Fondazione Bruno Kessler, Via Sommarive 18, 38123 Povo (TN), Italy.

[2]Fondazione Bruno Kessler, Via Santa Croce, 77, 38122 Trento, Italy.

[3]IULM University, Via Carlo Bo, 1, 20143 Milan, Italy.

[4]Berkman-Klein Center for Internet & Society, Harvard University, 23 Everett St # 2, Cambridge MA 02138 USA.

In Figures SM.1–SM.3 we display the same analyses as the ones conducted in Figure 4 in the main text. That is, we show the correlation between the degree and the sentiment, the latter being computed using the Big Five algorithm (Fig. SM.1), the Sentiments algorithm (Fig. SM.2) and the Wellbeing algorithm (Fig. SM.3). Each plot is accompanied by the robustness profiles, implemented with sentiment-based attacks. All these curves are qualitatively the same, independent of the algorithm employed to detect the sentiment.

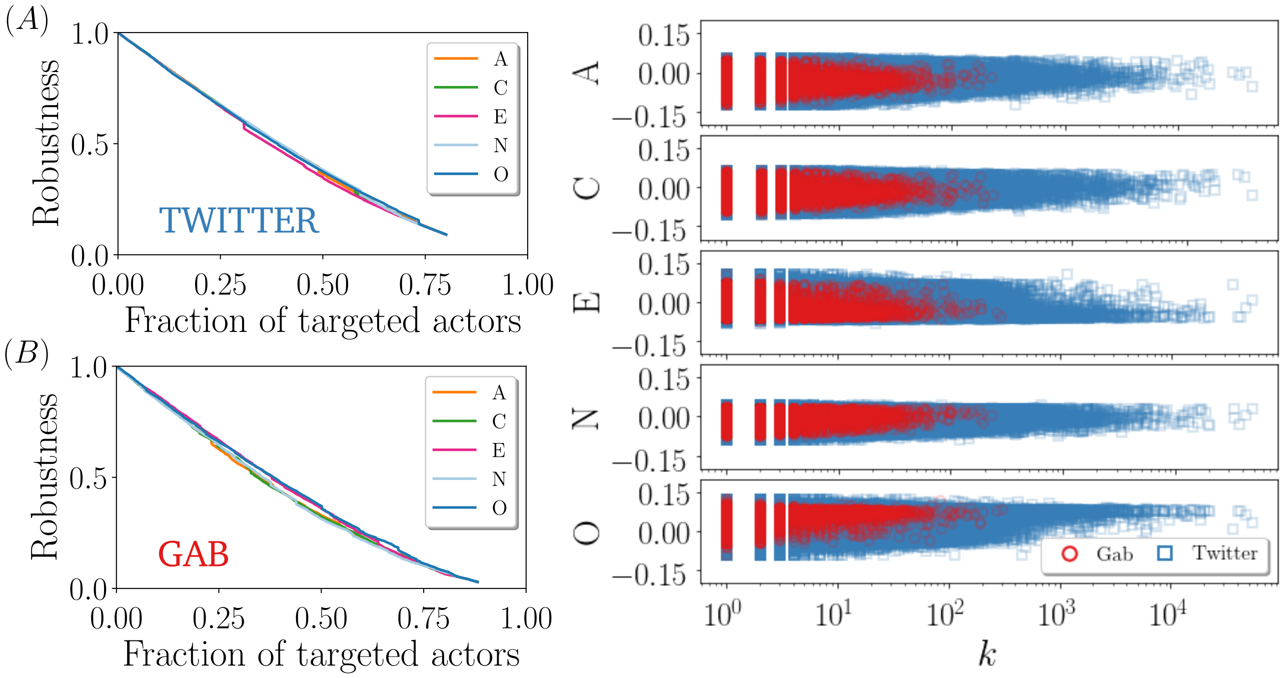

Figure SM.1: Size of the largest connected component for the sentiment-based attacks in Twitter and in Gab. Each curve corresponds to the sentiment targeted for the removal. On the right, the correlation between the degree of the nodes and the sentiment score computed by using the Big Five algorithm. The corresponding sentiment is written in the vertical axes. For the sake of clarity, in Twitter networks we only plot a random 20% selection of all users.

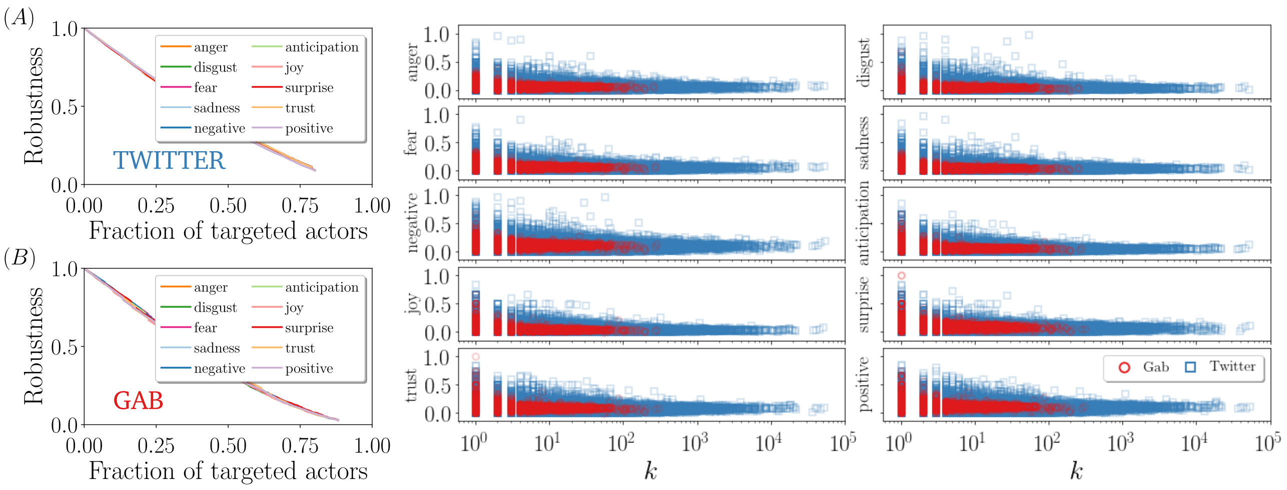

Figure SM.2: Size of the largest connected component for the sentiment-based attacks in Twitter and in Gab. Each curve corresponds to the sentiment targeted for the removal. On the right, the correlation between the degree of the nodes and the sentiment score computed by using the Sentiments algorithm. The corresponding sentiment is written in the vertical axes. For the sake of clarity, in Twitter networks we only plot a random 20% selection of all users.

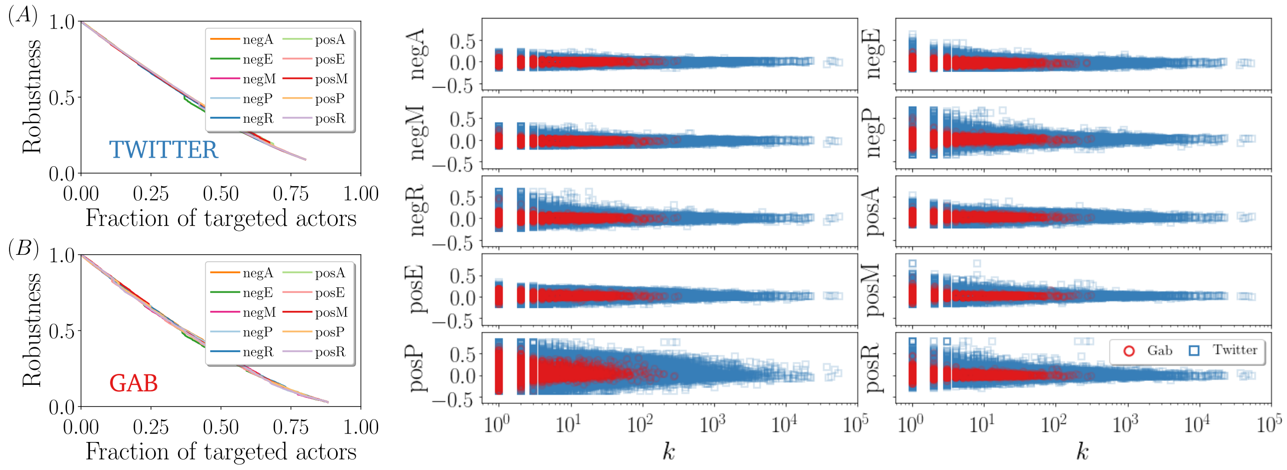

Figure SM.3: Size of the largest connected component for the sentiment-based attacks in Twitter and in Gab. Each curve corresponds to the sentiment targeted for the removal. On the right, the correlation between the degree of the nodes and the sentiment score computed by using the Wellbeing algorithm. The corresponding sentiment is written in the vertical axes. For the sake of clarity, in Twitter networks we only plot a random 20% selection of all users.
